# Incidental detection of prostate cancer with computed tomography scans

**DOI:** 10.1038/s41598-021-86972-y

**Published:** 2021-04-12

**Authors:** Steven Korevaar, Ruwan Tennakoon, Mark Page, Peter Brotchie, John Thangarajah, Cosmin Florescu, Tom Sutherland, Ning Mao Kam, Alireza Bab-Hadiashar

**Affiliations:** 1 School of Engineering, RMIT University, Melbourne, 3000 Australia; 2 School of Computing Technologies, RMIT University, Melbourne, 3000 Australia; 3 St Vincent’s Hospital, Radiology, Melbourne, 3000 Australia

**Keywords:** Computer science, Cancer imaging, Prostate cancer

## Abstract

Prostate cancer (PCa) is the second most frequent type of cancer found in men worldwide, with around one in nine men being diagnosed with PCa within their lifetime. PCa often shows no symptoms in its early stages and its diagnosis techniques are either invasive, resource intensive, or has low efficacy, making widespread early detection onerous. Inspired by the recent success of deep convolutional neural networks (CNN) in computer aided detection (CADe), we propose a new CNN based framework for *incidental detection* of clinically significant prostate cancer (csPCa) in patients who had a CT scan of the abdomen/pelvis for other reasons. While CT is generally considered insufficient to diagnose PCa due to its inferior soft tissue characterisation, our evaluations on a relatively large dataset consisting of 139 clinically significant PCa patients and 432 controls show that the proposed deep neural network pipeline can detect csPCa patients at a level that is suitable for incidental detection. The proposed pipeline achieved an area under the receiver operating characteristic curve (ROC-AUC) of 0.88 (95% Confidence Interval: 0.86–0.90) at patient level csPCa detection on CT, significantly higher than the AUCs achieved by two radiologists (0.61 and 0.70) on the same task.

## Introduction

Prostate cancer (PCa) is the second most frequent type of cancer found in men worldwide and the fifth leading cause of death by cancer. In 2018, there were 1,276,106 new cases and 358,989 deaths due to PCa^1^. If detected in its early stages, the survival rates of PCa are high due to slow progression of the disease^2,3^. Thus, with early detection and effective monitoring the odds of a patients’ survival may be improved.

There are a variety of clinically approved diagnostic methods for PCa including: prostate-specific antigen (PSA) tests, digital rectal examination, transrectal ultrasounds (TRUS), PSMA-PET scans, and magnetic resonance imaging (MRI). It has been identified that PSA determination in combination with transrectal ultrasound guided biopsy often leads to false-positive results and over-treatment^4^. The introduction of the Prostate Imaging Reporting and Data System (PI-RADS) in 2012 has led to a more standardized acquisition, interpretation, and reporting of prostate MRI^5,6^. Due to this, multi-parametric MRI which relies heavily on diffusion-weighted imaging (DWI) is increasingly becoming the standard of care for PCa diagnosis.

Prostate cancer generally does not present any symptoms until it becomes locally advanced or metastatic and thus can remain asymptomatic for many years^7^. This makes early diagnosis challenging. In the past, screening efforts have been focused on discovering these asymptomatic patients through PSA screening programs^4,8^. However, the extent of both benefits and harm of screening is under continuous debate as the risk of over diagnosis is substantial. Given this and the resource intensive nature of other diagnosis methods (biopsy and MRI), several countries, including Australia, do not run any prostate cancer screening programs^9^.

As current diagnosis methods are insufficient for incidental scanning and screening programs, exploring additional modalities could lead to the discovery of an effective tool for screening and early diagnosis for PCa. Computed Tomography (CT) imaging, for example, is more widely available than MRIs due to its speed and cheaper hardware. CTs have a high radiation dose which may lead to adverse effects in the long term meaning its use as a dedicated screening tool may not be justifiable. However, as CT scans are often taken for other reasons (where the adverse effects are already justified), using those for incidental screening may be viable. Unfortunately; in terms of prostate cancer, CT is currently considered insufficient in allowing humans to diagnose, localize, and monitor PCa due to its inferior soft tissue characterization^10^ compared to MRI.

Several recent studies have investigated the detection capacity of PCa in CT scans by expert radiologists. A study conducted by Jia et. al.^11^, on a limited cohort of 27 patients, showed that areas of focal mass-like enhancement on CT imaging directly correlates with prostate neoplasms (as revealed by multi-parametric MRI and follow-up targeted biopsy). A more recent study by Huang et al.^12^ investigated the use of contrast-enhanced CTs in detecting peripheral zone PCa using images from 100 patients with biopsy-proven PCa and 100 control subjects. They concluded that incidental detection of a focal area of increased enhancement in the periphery of the prostate may represent a clinically significant cancer and deserves further investigation with prostate-specific antigen measurement and correlation with other clinical risk-factors for PCa. While this is a finding in favour of using CT imaging to detect PCa; the research only shows results for cancer found within the peripheral zone which only accounts for 70% of cases. The above studies have shown the potential of using CT imaging in detecting PCa; but, further work is needed to address the small sample sizes, and limited type scope when assessing the usability of CT scans in detecting PCa.

While the above works show some promise in detecting PCa in CT imaging, training humans to find evidence of it consistently may be time consuming and expensive. By utilizing artificial intelligence (AI), along with a larger sample size of PCa cases, it may be possible to show whether PCa can be detected in CT scans or not and whether it can be used in a pipeline for incidental detection. Fortunately, there have been recent substantial improvements in AI systems being used to assist practitioners in medical image analysis tasks^13^. These systems which use deep convolutional neural networks (a subset of deep learning algorithms) have been gaining popularity in many fields. Several deep learning algorithms have been used successfully in computer aided detection (CADe) of PCa^14–20^. Furthermore, some studies have show that a Deep CNN model trained with T2-weighted and diffusion MRI achieves similar performance to clinical PI-RADS assessment^21^. However, these works have focused solely on multi-parametric MRI scans, and have overlooked other imaging techniques.

Inspired by the recent success of deep CNN models at detecting PCa in MRIs, in this paper, we investigate the use of deep CNNs for *incidental* detection of clinically significant prostate cancer (csPCa) in asymptomatic patients who had a CT scan of the abdomen/pelvis for another reason. Our hypothesis is that CNNs might be able to leverage subtle cues in CTs that are not visible to humans in order to boost csPCa classification performance. Our proposed CNN based pipeline for csPCa classification is trained end-to-end using patient level annotations. To the best of our knowledge, ours is the first work that use CT imaging in combination with a deep CNN for incidental detection of csPCa. A key challenge in training deep CNN models for medical image analysis is the limited availability of data. To overcome this issue, our proposed pipeline uses a combination of self-supervised feature learning techniques^22^ and data augmentation. We evaluate the performance of the proposed model on a dataset with 571 CT scans of the abdomen and pelvic region using cross-validation techniques and compare the models performance with the performance of expert radiologists on the same task.

The purpose of this study is two-fold: the first is to discover whether CTs are a possible avenue for prostate cancer detection and the second is to develop a fully automated deep learning pipeline capable of performing incidental detection of prostate cancer from CT scans. This pipeline is proposed to operate on all “at-risk” patients who undergo a CT scan, the automated pipeline would then signal an operator to perform further testing on patients with a high probability of having some form of prostate cancer. These further tests would remain unchanged from current diagnosis methods. Such an incidental screening tool would aid in the detection of prostate cancer that is in its initial asymptomatic phase, and if successful, has major implications for future incidental screening programs for other cancers.

## Methodology

This section describes how the CT data was collected and processed for use within a deep learning algorithm. In addition it gives the details of the 3D convolutional neural network pipeline: its structure, and its training and evaluation processes.

### Dataset

To train and validate our models, CT scans were collected from the electronic clinical database at St Vincent’s Hospital (Melbourne, Australia). To collect this data a set of criteria was formed. For an incidental screening program for prostate cancer the patients that need to be scanned are those at high-risk of prostate cancer: male and aged 50 years or older, which form the first two criteria. From here two different sets of scans will be collected: one set of scans with PCa, and one as a control set of “healthy” prostate scans.

The “PCa positive” scans will be of male patients, older than 50 years, that have been diagnosed with clinically significant PCa (csPCa) (i.e., a Gleason Score of $3+4$ or higher, or an International Society of Urological Pathology score of 2 or higher) confirmed with either a transrectal ultrasound-guided biopsy or prostatectomy.

While this criteria leads to a very broad range of lesion sizes this accurately reflects the natural variation in prostate cancer lesions that need to be detected by an incidental screening program. As we do not aim to differentiate between different Gleason scores, no controlling for those scores has taken place. From each csPCa patient, the chosen CT scan will be the closest scan to the biopsy (a maximum of 6 months prior, or 6 weeks after the biopsy to allow the prostate to heal visible signs (with a maximum of 3 months post biopsy).

Likewise, control scans will be from male patients, 50 years or older, that have no prior history or suspicion of PCa at the time of data collection. Given the prevalence of PCa in the general population, some of the control subjects may have some level of PCa that was not diagnosed by the time of this study.

Using the above selection criteria 571 scans were collected, 139 had clinically significant prostate cancer, and the remaining 432 were control cases. Due to the selection criteria, We have no reason to suspect that this sample will be systematically different from the population of scans that an incidental screening tool would be applied to in the future.

All scans were captured between 2013 and 2020. The age range of the subjects were 50–103 and had a mean age of 71.6 (25th and 75th percentile ages were at 62 and 80, respectively).

The process for diagnosing and scanning a patient with possible PCa begins with a clinical examination, then MRI scans to check for signs of visible PCa. The PCa is then confirmed using ultrasound-guided biopsy or a prostatectomy depending on severity. If there the urologist is very suspicious based on the clinical examination then a biopsy may be performed prior to imaging, though this is rare. To minimize any post-biopsy changes in the prostate scans were collected 6 weeks post-biopsy to give adequate time for healing.

For all patients, the scans were obtained with 64-MDCT scanners (either a Siemens SOMATOM or GE revolution) in 5mm-thick axial sections using dual energy (unless the patient was larger than 320cm). All examinations were performed with the patient performing a breath-hold and the following parameters: thin slice, 1mm for Siemens SOMATOM and 1.25mm for GE revolution; 100ml of Optiray 350 injected IV at 2.5ml/s during the venous phase (70s delay); Exposure, 70-140kV, 120 mAs.

The evaluations were done using five-fold cross validation. Each fold was selected to hold the same ratio of positives to controls (clinically significant PCa vs no known PCa) by splitting the positives and controls into five subsets separately, 28 positives and 86 controls per fold. Given there were no-identified confounding elements, beside positive and negative ratios, within our dataset that would require controlling when selecting a sub-sample. Random sampling should yield a representative sample of the total population. Given the entire dataset is used for validation the results should be representative of the total study group.

#### Ethics

The Research Governance Unit of St Vincent’s Hospital Melbourne approved this retrospective study in accordance with the research conforming to the National Health and Medical Research Council Act 1992 and the National Statement on Ethical Conduct in Human Research 2007 (updated July 2018). Project ID: 68554. St Vincent’s Local Reference Number: LRR 231/20.

All methods undertaken in this project were carried out in accordance with the above ethics approval. The above ethics approval has waived the requirement for informed consent.

All methods were performed in accordance with relevant regulations and guidelines.

#### Data preprocessing

Before any pre-processing, the CT scan’s dimensions are roughly $512 \times 512 \times 300$ voxels and contain the entire body’s cross-section. To reduce the amount of unrelated data presented to the AI model and to provide a fixed sized input as required by the neural networks, each CT scan was cropped to the size $150 \times 150 \times 14$ (in the coronal, sagittal, and axial axes, respectively), this size is the smallest size which captures the whole prostate. However, in some cases where the prostate is small, there is a significant amount of data surrounding the prostate that is included in the input. Next, the voxel values were clipped to the range $\left[ -250 \text {HU}, 500 \text {HU} \right]$ to capture 95% of the variation in values within the prostate region and to give more contrast to the softer tissue. Finally the voxel values were normalized to the range $\left( 0, 1 \right)$ using the min-max normalization method. Here for each scan ${\mathbf {X}}_i$ the normalized scan, $\hat{{\mathbf {X}}}_i$ was computed as:

1 $$\begin{aligned} \hat{{\mathbf {X}}}_i = \frac{{\mathbf {X}}_i- 500}{500 + 250} \end{aligned}$$

**Figure 1:**
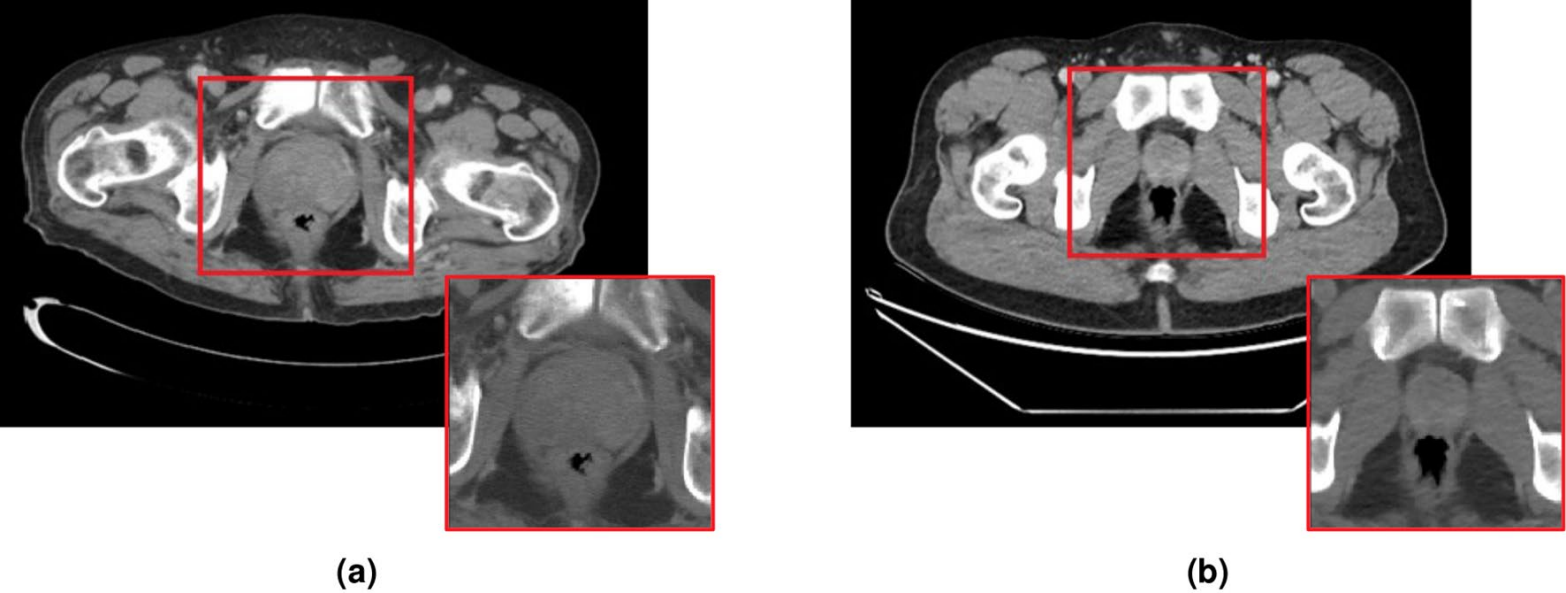
Two CT scan slices from the dataset: (**a**) from a confirmed csPCa patient, and (**b**) from a patient with no known prostate cancer (control). Both include a red square indicating the cropped prostate region, and the pre-processed version on the lower right corner.

Figure 1 shows two CT scan slices and the corresponding pre-processed slices: one from a patient with confirmed csPCA 1a, and one from a control patient 1b.

In our experiments we compare the performance of the proposed CNN pipeline to radiologists on csPCA detection with CT. For this purpose we developed an in-house visualization interface which takes a CT image from the dataset and presents it to a radiologist. When presenting the information to the radiologist, two modifications were made to pre-processing which were not required for the machine learning pipeline: **(1)** The CT images shown to the radiologists consisted of 20 CT slices around the prostate instead of 14 (at request of the radiologists as several scans had 1 or 2 slices missing from the top of the prostate on larger than average-sized prostates). **(2)** The voxel normalisation was adjusted to provide more contrast for humans to see details in the prostate (range $\left[ -360 \text {HU}, 440 \text {HU} \right]$).

### Pipeline

**Figure 2:**
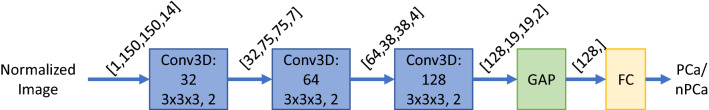
The 3D convolutional neural network pipeline used for classification. The input is a pre-processed CT scan of $150 \times 150 \times 14$ voxels, and the output is a single probability (from 0 to 1) of having csPCA.

We use a deep convolution neural network to classify the CT scans into csPCA vs control. A deep CNN learns a non linear function ($f_\theta : {\mathbb {R}}^{150\times 150\times 14} \rightarrow \left\{ 0, 1\right\}$) that takes in a CT scan ${{\mathbf {X}}}_i$ and maps that to a value ${\hat{y}}_i$ in the range $\left( 0, 1\right)$ depending on whether the input CT scan is from a csPCA patient (${\hat{y}}_i$ = 1) or a control patient (${\hat{y}}_i$ = 0). The CNN architecture used in our pipeline consists of three 3D-convolution layers each with convolution filters of the size $3 \times 3 \times 3$ with a stride of 2 to downscale the input, a batch normalization layer, and ReLU activation function. The number of filters in each of those convolution layers were 32, 64, and 128. The output of the third convolution layer was then subjected to Global Average Pooling (GAP). Finally, a fully connected layer with Sigmoid activation was used as the classifier. Figure 2 shows the structure of the CNN model used for classification and supervised training.

### CNN training

Given a training dataset ${\mathscr {D}} = \left\{ \left( \hat{{\mathbf {X}}}_i, y_i \right) \right\} _{i=1}^N$, with *N* CT scans and the associated ground truth labels $y_i \in \left\{ 0, 1\right\}$, we can use supervised learning techniques to obtain the parameters (weights) of the above CNN model. The loss function used in training the proposed CNN model was the following weighted cross entropy loss function:

2 $$\begin{aligned} {\mathscr {L}}_{ce}\left( \theta \right) = \sum _{i=1}^N \left( w_1 y_i \log \left( f_\theta \left( \hat{{\mathbf {X}}}_i\right) \right) + w_0 \left( 1-y_i \right) \log \left( 1 - f_\theta \left( \hat{{\mathbf {X}}}_i\right) \right) \right) + \lambda \left\| \theta \right\| _2^2. \end{aligned}$$

The term $w_i$, is the balancing weight for class *i* and used to make the loss contribution from each class equal, is computed as:

3 $$\begin{aligned} w_i = \frac{\left| {\mathscr {D}}_i \right| }{\left| {\mathscr {D}} \right| } \end{aligned}$$

where $\left| {\mathscr {D}}_i \right|$ is the number of CT scans from class *i* in the training set ($i=0$ for the control class and $i=1$ for csPCA). The hyper-parameter $\lambda$ is a weight regularization coefficient (called weight decay in the AdamW optimizer used for training) that controls the complexity of the model preventing it from under/over fitting.

It is known that training a deep CNN models from random initialization requires a large amount of data. However, as mentioned in the dataset section, we only have a relatively small number of data (for each cross validation fold we have 452 CTs for training and 113 CTs for validation). In deep learning, both data augmentation and self-supervised pre-training techniques can be used to create a more robust (and thus more general) model when only a small set of labelled training data is available. Therefore, a variety of augmentation and self supervised pre-training techniques have been applied in training the above CNN model.

In deep learning, data augmentation is a technique that transforms training inputs in a way that does not impact the classification label of the sample (rotations, translations, small amounts of noise, etc). This makes the model become more robust and generalize to samples outside of the training set distribution. For this pipeline, the input (pre-processed) CT scans are subjected to random rotations and translations during the training process. A rotation angle for each image in a given epoch was randomly selected from the set $\left\{ 0^{\circ }, 90^{\circ }, 180^{\circ }, 270^{\circ }\right\}$ whereas, the translations are sampled uniformly from the range [-25, 25] and [-2, 2] for height/width and depth dimensions, respectively.

In self-supervised learning, a CNN is first trained on a pretext task for which the labels can be generated through some automated process. The knowledge gained in doing this pretext task is then used as a starting point for learning the desired (down-stream) task; in our case csPCA / control classification. The objective of self supervised learning is to leverage unlabelled data to build a feature representation that will be useful for the down-stream task. The most common self-supervised pretext tasks are rotation discrimination^23^, image in-painting^24^, or reorder sections of an image to their correct location^23^.

**Figure 3:**
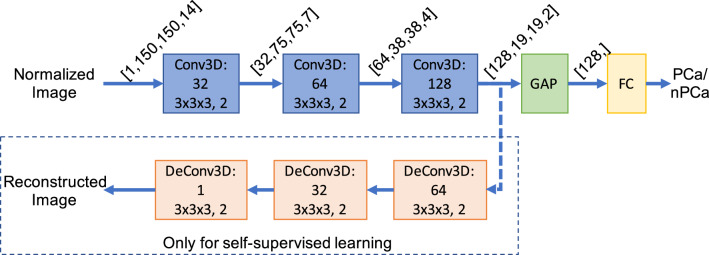
The CNN pipeline with the additional reconstruction branch for the patch-swap reversal self-supervised pre-training. For patch-swap reversal training the input is a pre-processed and corrupted (see patch swap reversal section for details) CT scan of $150 \times 150 \times 14$ voxels, and the output is the reconstructed pre-processed CT scan of the same size.

In the proposed pipeline, we used self-supervision to pre-train the convolution stages of the network in Fig. 2. Patch-swap reversal was selected as the pretext task (the details of which are given below). To allow the network to perform the patch-swap reversal task, a deconvolution branch was added after the initial encoding. This branch has the same structure as the encoder but reversed (convolution layers replaced by transpose convolutions); which reconstructs the full-sized scan from the compressed version. The CNN structure with the deconvolution branch used for the self-supervised pre-training can be seen in Fig. 3.

#### Context reconstruction (patch swap reversal)

For a pretext task to yield a benefit in a downstream task, it must be able to train a network to build a strong and generalizable understanding of the dataset. One common pretext task, in-painting, requires a strong understanding of a dataset to be able to reconstruct missing data within a sample; however, as missing data is replaced with a constant value, identification of corrupt sections is too simple. A new task, patch-swap reversal, has been proposed in an attempt to rectify this issue, and has been shown to be useful in the medical domain already^24^. The main advantage and distinction in patch-swap reversal is that it maintains the same image intensity distribution throughout the corruption process as opposed to in-painting, meaning the network must have a greater understanding of the dataset to identify corrupted patches before it can correct them.

**Figure 4:**
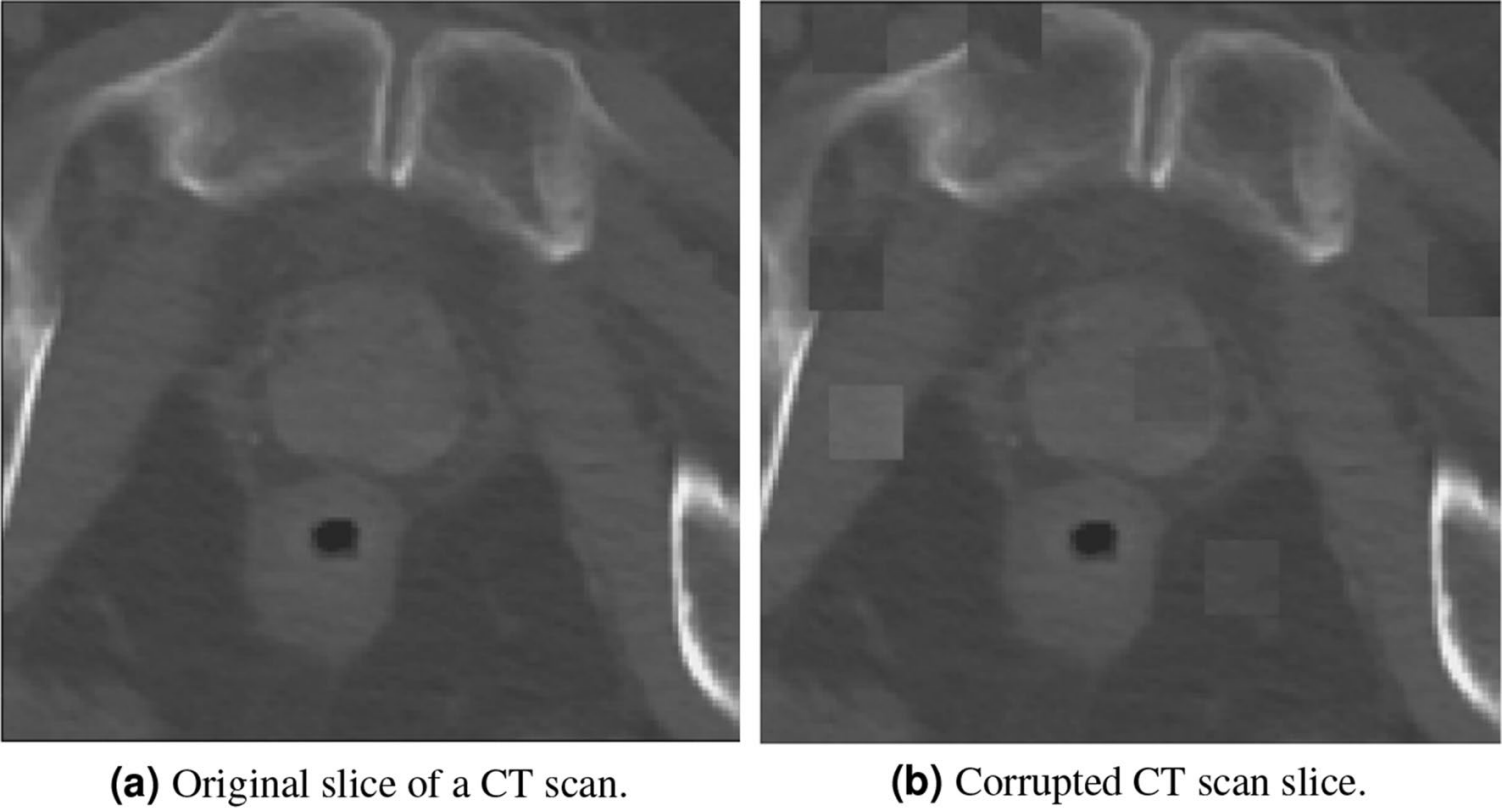
Examples of a single slice from the patch swap reversal pretext task.

In patch swap reversal, for each training image, $X_i$, a corrupted version of that image, ${\hat{X}}_i$, is generated by: randomly selecting two small patches in the original image and then swapping the voxel values between them. This swapping process is repeated for a predetermined number of times on each image. In our experiments we set the patch size to $20 \times 20 \times 3$ with 15 swap operations for each image (for a total of 30 patches). An example CT scan before and after the patch swap reversal process is shown in Fig. 4. Once the dataset, $\left\{ \left( X_i, {\hat{X}}_i\right) \right\} _{i=1}^N$, is generated the network is trained to reconstruct the original image $X_i$ using the corrupted version of it ${\hat{X}}_i$. The loss function for the pretext task was the mean squared error (L2 norm) between the reconstructed image and the original image.

4 $$\begin{aligned} {\mathscr {L}}_{L2} = \frac{1}{N} \sum _{i=0}^{N} \left\| X_i - f_{\theta _2} \left( {\hat{X}}_i \right) \right) \Vert ^2_2 \end{aligned}$$

The model was trained using the AdamW optimizer. First it trained on the pretext task with learning rate 3e-4 for 60 epochs, then trained for a further 100 epochs with a learning rate of 3e-5 on the csPCA vs control classification (down-stream) task. Throughout the training process the regularization parameter $\lambda$ was kept at 3e-5, which was found through manual parameter searching on a subset of data.

## Results

In this section, we evaluate the performance of the proposed CNN pipeline on CT based prostate cancer classification and compare it with the performance of radiologists (on the same task). We use Receiver Operating Characteristics (ROC)^25^, sensitivity, specificity and Cohen’s Kappa^26^ matrices, computed via five-fold cross validation, to quantify the performance of the model in separating confirmed csPCA and control cases. All experiments were executed in PyTorch on a computer with Intel Xeon E5-1650 CPU with an NVIDIA GTX Titan X GPU, 16GB RAM and Ubuntu 16.04 OS.

First, the contribution of each element of the proposed pipeline was identified using an ablation study. Here, we trained models using four different variants of our pipeline:

**Table:** 

$\bullet$ No data augmentation or self supervision	$\bullet$ Only self supervision (no data augmentation)
$\bullet$ Only data augmentation (no self supervision)	$\bullet$ Both data augmentation and self supervision (proposed method).

The results of the ablation study is presented in Table 1. The results show that the model trained without augmentation or self supervision was only able to achieve a ROC-AUC of approximately 0.82. Adding data augmentation and self supervision independently, improved the performance of the model to around 0.845 and 0.842, respectively. The model trained with both data augmentation and self-supervision was able to achieve significantly better performance (ROC-AUC $\approx 0.877$). The above results show that both data augmentation and patch-swap reversal based self-supervision can help the training process to converge to a model that generalizes well to test data.

**Table 1:** The results from the ablation study: the mean and 95% Confidence Interval of the area under the ROC curves measured using 5-fold cross validation.

	No pre-training	Patch swap reversal SSL
No augmentation	0.821 (95% CI: 0.79–0.86)	0.842 (95% CI: 0.80–0.88)
With augmentation	0.845 (95% CI: 0.82–0.87)	**0**.**877** (**95%** **CI:** **0**.**86**–**0**.**90**)

The bold value indicates the highest accuracy permutation of the pre-training and augmentation variations.

Next, we compared the performance of the model on classifying prostate cancer on CTs with the performance of human experts (i.e. radiologists). Thus, for this experiment, we randomly selected 115 (or $20\%$) CT scans from the original dataset (one of the five folds used in the cross validation) that consisted of 28 scans from confirmed csPCA patients and 87 control patients. This data subset was then presented to two expert radiologists with 7 and 10 years of experience respectively. The radiologists examined the CT scans and annotated each scan with two labels: 1) Does the scan contain indications of csPCA (or not), and 2) A confidence level (from 0 to 5) quantifying their confidence in making the decision. In order to compute the AUC values, the annotations by the radiologist’s were converted to probabilities using the following procedure. If the radiologist had the highest confidence level (5) and had found no prostate cancer this would be considered a score of 0; likewise, if they had found prostate cancer with high confidence it would be a score of 1. If the radiologist had zero confidence, regardless of whether prostate cancer was found or not the score would be 0.5. The values between were obtained using linear interpolation on confidence level; lower confidence means the score is closer to 0.5.

**Figure 5:**
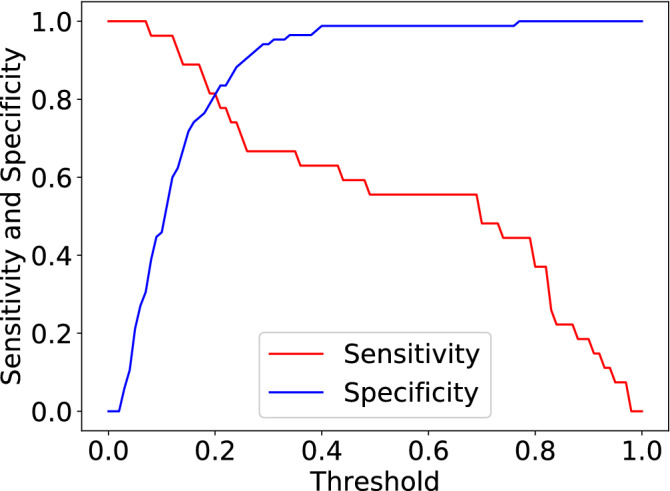
Sensitivity and Specificity Curves of the Proposed CNN Pipeline with respect to the chosen threshold.

Table 2 compares the performance of the proposed CNN model with the expert radiologists. The results show that the ROC-AUC for both radiologists are between 0.61-0.70. Radiologist 1 inclined towards achieving a high specificity while Radiologist 2 inclined towards achieving a high sensitivity. In comparison, the proposed CNN model has achieved a significantly higher ROC-AUC ($\approx 0.87$) with a specificity of 0.99. When calculating the sensitivity/specificity for the proposed CNN model, we used a threshold of 0.5 which gives a high specificity but low sensitivity of 0.56. For most applications a higher sensitivity is desired^27^, but in incidental detection applications it can be argued that a higher specificity is useful to push uncertain cases towards further testing. If a higher sensitivity is required, one can set the threshold to 0.2 which will give both a sensitivity and specificity of $\approx 0.8$, the full spectrum of sensitivities and specificities for the complete dataset is shown in Fig. 5.

**Table 2:** Performance comparison between proposed CNN pipeline and two radiologists on PCa classification with CT scans.

	Proposed CNN pipeline	Radiologist 1	Radiologist 2
ROC-AUC	**0**.**877**	0.613	0.697
Sensitivity	0.556	0.444	**0**.**741**
Specificity	**0**.**988**	0.671	0.600

The bold element in each row indicates the strongest performing model for that row’s metric (ROC-AUC, sensitivity, and specificity).

**Table 3:** Cohen’s Kappa Agreement between the proposed CNN pipeline, radiologists, and ground truth labels.

	Ground-truth labels	Proposed CNN pipeline	Radiologist 1
Proposed CNN pipeline	**0**.**632**	–	–
Radiologist 1	0.086	0.107	–
Radiologist 2	0.254	0.157	0.191

The bold element indicates the highest agreement between raters.

Table 3 shows the Cohen’s Kappa Agreement between the proposed CNN model, radiologists, and the ground truth labels. Cohen’s Kappa is a measure between $(-1, 1)$ where a value of 0 indicate no agreement and a value of $+1$ indicate perfect agreement and $-1$ indicates perfect disagreement. An interpretation for Cohen’s Kappa values in the context of medical data is provided by McHugh^28^ (see Table 3). The results show that the agreement between the proposed CNN model and ground truth labels is moderate. However, the two radiologists showed minimal to no agreement with the ground truth labels. Furthermore, the agreement between the two radiologists in csPCA scan classification is also minimal.

The above results, inline with the previous observations, show that for *humans*, CT scans do not provide adequate information to classify csPCA accurately^10^. Our results are lower compared to the observations made by Huang et al.^12^ who showed humans were able to identify peripheral zone cancer with a high sensitivity and specificity (0.83 and 0.92, respectively). There are several differences between Huang et al.’s work and this: most notably Huang et al. included clinically non-significant PCa (with Gleason scores of 3+3 and ISUP 1 or higher), as well as excluding cases with severe prostatomegaly, extensive calcification, and hip replacements, and cases of PCa that were within the transition zone. Overall, these exclusions make the task of detection of PCa in CTs easier, and are less representative of daily clinical practice as opposed to a non-curated set of samples. Similarly to Huang et al., the radiologists in our experiments, routinely review the prostate on CT scans. However it is often difficult to identify small or early prostate cancer and therefore in daily clinical practice they would raise the possibility of prostate cancer when they identify cases with large enhancing tumours, when the tumour extends beyond the prostate or when they identify lymph node and bone metastasis.

The proposed CNN model has been able to utilize the information in CT scans to classify clinically significant PCa cases vs cases with no known PCa at a level that is deemed adequate for incidental detection of PCa.

**Figure 6:**
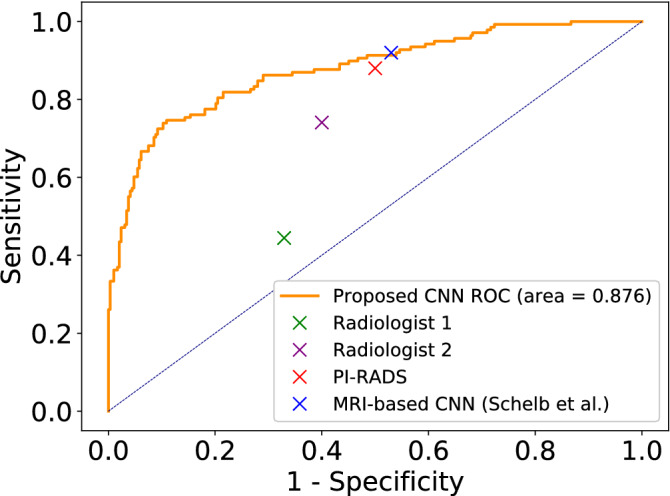
ROC Curve of the proposed CNN model in comparison to the sensitivity and specificity of the two radiologists, PI-RADS, and deep learning systems on MRIs by Schelb et al.

## Discussion

As discussed in the introduction, several recent studies have used deep neural network based methods to diagnose PCa in MRI. For example, Schelb et al.^21^ compared the PCa classification performance of a deep convolutional neural network (CNN) model (trained on T2-weighted and diffusion weighted MRI (DWI)) versus clinical PI-RADS assessment. The PI-RADS had a sensitivity of 88% at specificity of 50% whereas the CNN model had a sensitivity of 92% with specificity 47%. The results show that a CNN model trained with T2-weighted and diffusion MRI achieves similar performance to clinical PI-RADS assessment. More recently, Yoo et al.^18^ used a dataset of 427 (175 patients with PCa and 252 patients without PCa) DWI scans with slice level expert annotations to train a deep convolutional neural network (CNN) model to detect PCa. Their pipeline, achieved an area under the receiver operating characteristic curve (AUC or ROC-AUC) of 0.84 (95% confidence interval: 0.76–0.91) for patient level PCa detection.

Figure 6 shows an overview of the comparison of the proposed CNN pipeline with the reported performance of MRI based prostate cancer detection with PI-RADS and deep CNN based models, and humans observing CTs. The values for PI-RADS and the deep CNN models were obtained from Schelb et al.^21^. We acknowledge the fact that these values were obtained on different datasets and are not directly comparable. We do not claim that the proposed pipeline has comparable diagnostic capability to MRI based methods, our intention here is to show that the proposed pipeline had adequate performance level to be used for incidental detection.

According to Schelb et al.^21^, PI-RADS has a sensitivity 0.88 at specificity of 0.5 and the deep CNN model operating on MRIs had a sensitivity of 0.92 with specificity 0.47; these models place more priority on detecting positive csPCA cases than finding control samples. Figure 6 shows that the proposed CT deep learning model performs similarly to PI-RADS and other MRI-based deep learning model at equivalent sensitivities / specificities. In terms of AUC: similar deep learning systems operating on MRIs (proposed by Yoo et al.^18^) achieved AUCs of approximately 0.85 compared to our 0.88 on CTs. The small variations in the AUC values can likely be attributed to the use of strong data augmentation, self-supervised pre-training or different difficulty level of the datasets used. These results comparisons further indicate that CTs do hold information that is useful for classifying prostate cancer.

A significant limitation of our pipeline is the inability for the model to provide localisation information for the cancer. However; in comparison to PSA tests (another diagnosis technique that gives a probability and no localisation information), our pipeline has superior performance. According to prior research: when using a threshold of 3 ng/mL PSA tests have a sensitivity of 0.32 and specificity of 0.85^29^ compared to this pipeline achieving a sensitivity and specificity of 0.56 and 0.99 respectively.

**Figure 7:**
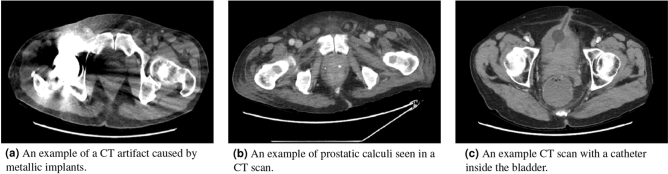
A set of examples showing different artifacts that were found within the dataset: metallic objects causing spikes of noise, calculi within the prostate, and catheters.

We also conducted a qualitative evaluation of the proposed CNN based pipeline. For this evaluation we extracted strong false positives, false negatives, true positives, and true negatives from the model and showed them to an expert radiologist. Many of the false positive samples had other issues within the scan that were not prostate cancer; such catheters, and prostatic calculi (as seen as a small white dot in the prostate). Some examples can be seen in Fig. 7. Likewise, some of the false negatives were of scans with insufficient contrast level. This shows that the CNN model may not be robust to unseen types of anomalies in the data. Such issues can be avoided by either including more data with anomalies in the dataset or by doing an image quality classification before the images are fed to the CNN model. However, for the strong true positives no trend was seen, giving some evidence that there is no confounding element that the CNN identifies as PCa incorrectly.

## Future work

While this study has been successful there are many areas which will need to be improved on in future work. The main are that must be explored is to gather a wider variation of data and controlling for different factors. Our current dataset is limited as it is sourced from a single hospital, and does not differentiate between cancer locations or cancer severity. Future work should be aimed at assessing this pipeline’s performance in detecting different variations of cancer. Likewise, extensive research needs to be done in assessing healthcare outcomes of patients when such a tool is used in practice.

## Conclusion

In this work a 3D convolutional neural network based pipeline was developed for incidental detection of prostate cancer patients using contrast enhanced CT scans. An incidental screening tool for prostate cancer has the potential to improve health outcomes significantly as it could prompt further testing of patients who are in the long initial asymptomatic phase of the cancer. The model was evaluated by performing five-fold cross validation on a dataset of 571 CT scans (139 scans from clinically significant prostate cancer patients confirmed either via transrectal ultrasound–guided biopsy or MRI and, 432 control patients with no known prostate cancer) along with a comparison to human radiologists on the same dataset and similar pipelines operating on MRIs. The proposed pipeline achieved an area under the receiver operating characteristic curve (ROC-AUC) of 0.88 (95% Confidence Interval: 0.86–0.90) at patient level PCa detection on CT which was significantly better than the human performance on the same task. Likewise, the results also show that CT in combination with deep learning can perform comparably to MRI based diagnostic pipelines, however future work that directly compares MRI and CT under the same settings is needed to confirm this. Overall, these results show that CT scans do contain enough information to separate PCa scans from non-PCa scans and thus gives a strong promise for real world applications of the pipeline in incidental detection of prostate cancer using CT scans and could lead to similar developments for cancers.

This study has shown that CT scans contain information to enable detection of prostate cancer, detectable using our deep learning pipeline. The proposed self-supervised deep learning pipeline holds promise in being applied to similar tasks in other areas for stronger models; however, this must be shown further.

## Data Availability

The dataset used during this study may be made available on request from the authors upon approval for extended distribution of the data by St Vincent’s Hospital and RMIT University.
